# Platinum Atoms and Nanoparticles Embedded Porous Carbons for Hydrogen Evolution Reaction

**DOI:** 10.3390/ma13071513

**Published:** 2020-03-26

**Authors:** Jialing Kang, Mengjia Wang, Chenbao Lu, Changchun Ke, Pan Liu, Jinhui Zhu, Feng Qiu, Xiaodong Zhuang

**Affiliations:** 1 School of Chemical and Environmental Engineering, Shanghai Institute of Technology, Shanghai 201418, China; 176061210@mail.sit.edu.cn; 2 The Meso-Entropy Matter Lab, State Key Laboratory of Metal Matrix Composites, Shanghai Key Laboratory of Electrical Insulation and Thermal Ageing, School of Chemistry and Chemical Engineering, Shanghai Jiao Tong University, Shanghai 200240, China; castle@sjtu.edu.cn; 3 School of Materials Science and Engineering, Shanghai Jiao Tong University, Shanghai 200240, China; wmj10063@sjtu.edu.cn (M.W.); panliu@sjtu.edu.cn (P.L.); 4 School of Mechanical Engineering, Shanghai Jiao Tong University, Shanghai 200240, China; kechangchun@sjtu.edu.cn

**Keywords:** platinum, atomically dispersed active site, nanoparticle, porous carbon, hydrogen evolution reaction

## Abstract

Due to the growing demand for energy and imminent environmental issues, hydrogen energy has attracted widespread attention as an alternative to traditional fossil energy. Platinum (Pt) catalytic hydrogen evolution reaction (HER) is a promising technology to produce hydrogen because the consumed electricity can be generated from renewable energy. To overcome the high cost of Pt, one effective strategy is decreasing the Pt nanoparticle (NP) size from submicron to nano-scale or even down to single atom level for efficient interacting water molecules. Herein, atomically dispersed Pt and ultra-fine Pt NPs embedded porous carbons were prepared through the pyrolysis of Pt porphyrin-based conjugated microporous polymer. As-prepared electrocatalyst exhibit high HER activity with overpotential of down to 31 mV at 10 mA cm^−2^, and mass activity of up to 1.3 A mg_Pt_^−1^ at overpotential of 100 mV, which is double of commercial Pt/C (0.66 A mg_Pt_^−1^). Such promising performance can be ascribed to the synergistic effect of the atomically dispersed Pt and ultra-fine Pt NPs. This work provides a new strategy to prepare porous carbons with both atomically dispersed metal active sites and corresponding metal NPs for various electrocatalysis, such as oxygen reduction reaction, carbon dioxide reduction, etc.

## 1. Introduction

With the advent of smart cities, people have an increasingly high demand for new energy, and traditional fossil energy sources, such as coal and petroleum, can no longer meet the demand. One is the energy shortage caused by the excessive consumption of traditional fossil energy; the other is that a large amount of carbon dioxide (CO_2_) will be generated during the use of traditional fossil energy, which leads to the rising level of CO_2_ in the atmosphere and cause irreversible damage to the global ecosystem. In order to solve these problems, new energy and carriers need to be effectively used. New energy sources (such as solar, wind, and tidal energy) discovered by mankind have been vigorously developed because of their cleanliness and sustainability. Among them, hydrogen energy is an ideal alternative to the conventional fossil energy because the clean nature of hydrogen oxidation without greenhouse gas production, and ultra-high gravimetric energy density (120 MJ kg^−1^) [1,2]. Since Boddy et al. reported the first scientific case on water splitting by using n-type semiconductor TiO_2_ as anode material in 1968 [3], many researchers have been devoted to the study of hydrogen production by water splitting [4,5,6,7,8,9]. Conventional hydrogen production methods are mainly based on fossil-fuel technology, such as steam reforming [9], partial oxidation and coal gasification [10,11], which are not sustainable and usually lead to heavily CO_2_ emission. Besides, nuclear energy [12] and thermogravimetry analyzer (TGA) [13] technologies both can be used to produce hydrogen. In contrast, the production of hydrogen from renewable resources, such as solar [14,15], wind [16], water [17] and biomass [18] have achieved zero emission. Water electrolysis is a mature technology, accounting for about 4% of the world’s hydrogen production. It is the process of combining water molecules with electricity to decompose water into pure hydrogen and pure oxygen [17]. Therefore, hydrogen from water splitting is more favorable in view of its relatively long history and environmental protection characteristics. As a result, more and more researchers have focused on the exploration of high efficient electrocatalysts for hydrogen evolution reaction (HER) [19,20]. So far, platinum (Pt)-based electrocatalysts are the benchmark for HER [21,22,23,24], because of their superior performance, although many kinds of electrocatalysts, such as metal alloys [25], oxides [26], carbides [27], nitrides [28], phosphides [29], and carbon-based nanomaterials [30], etc., have been developed. However, the commercial application of Pt-based electrocatalysts is hindered by complicated preparation procedures and relatively poor long-term stability [31,32]. Hence, to improve the performance of Pt-based electrocatalysts for HER remains great challenge.

Several strategies have been discovered to improve the performance of Pt-based electrocatalysts, e.g., alloying with transition metal (nickel, cobalt, iron, etc.) [33,34], controlling the morphology with special size and shape (sphere, cube, polyhedron, nanowire, nanorod, nanotube, etc.) [35,36,37,38,39], diminishing the geometric size from submicron to nano-scale or even down to single atom level, etc. [40,41,42]. Many results demonstrate that Pt clusters or single atoms exhibit comparable or even superior performance than commercial Pt/C, such as Song et al. anchored Pt atoms onto onion-like carbon nanosphere with high HER performance (overpotential of 38 mV at 10 mA cm^−2^) [2]; Li et al. prepared Pt single atoms direct from bulk Pt by a thermal emitting strategy for HER (overpotential is only 23 mV at 10 mA cm^−2^) [23]. However, rational synthesis of Pt-based materials with controllable particles/clusters and single atoms is still a great challenge because of the agglomerate nature of Pt. One of the most efficient methods to stabilize the Pt clusters and single atoms is anchoring them by heteroatoms, like nitrogen (N). The interaction and electron transfer between Pt atoms and heteroatoms could synergistically enhance the activity and stability of as-prepared electrocatalysts [43,44].

In this work, Pt single atoms and Pt ultra-fine NPs embedded porous carbons were prepared by directly pyrolysis of Pt porphyrin-based conjugated microporous polymer. The atomically dispersed Pt and ultra-fine Pt NPs can be obviously observed through high-angle annular dark-field scanning transmission electron microscopy (HAADF-STEM). As electrocatalysts for HER, as-prepared hybrid materials exhibit overpotential of down to 31 mV at 10 mA cm^−2^ and mass activity of up to 1.3 A mg_Pt_^−1^ at overpotential of 100 mV, which is twice that of commercial Pt/C (0.66 A mg_Pt_^−1^). Such promising performance can be ascribed to the high electric conductivity of carbon matrix and synergistic effect of atomically dispersed Pt and ultra-fine Pt NPs. This work provides a new strategy to prepare porous carbons with both atomically dispersed precious metal active sites and corresponding metal NPs for energy conversion.

## 2. Materials and Methods

### 2.1. Materials

Pyrrole, p-bromobenzaldehyde, nitrobenzene, acetic acid, platinum dichloride (PtCl_2_), benzonitrile (PhCN), sodium propionate, dichloromethane (CHCl_2_), bis(1,5-cyclooctadiene) nickel(0) [Ni(cod)_2_], 2,2′-bipyridine and carbon black were purchased from Aladdin Reagent (Shanghai, China). Methanol (CH_3_OH), chloroform (CHCl_3_), acetonitrile, silica gel (100–200 mesh), chlorobenzene, triethylamine, 1,5-cyclooctadiene (cod), anhydrous N, N-dimethylformamide (DMF) and tetrahydrofuran (THF) were bought from Titan (Shanghai, China). Sulfuric acid (H_2_SO_4_, 98 wt. %) and hydrochloric acid (HCl, 36 wt. %) were obtained from Sinopharm Chemical Reagent (Shanghai, China). Pt/C (20 wt. %) were purchased from Sigma-Aldrich (Shanghai, China). Nafion solution (0.5 wt. %) was purchased from DuPont, Ltd (Shanghai, China).

### 2.2. Electrocatalysts Synthesis

#### 2.2.1. Synthesis of 5,10,15,20-tetrakis(4-bromophenyl)-21,22-dihydroporphyrin (TPP4Br)

*p*-Bromobenzaldehyde (7.5 g, 40 mmol) was dissolved in the mixed solvent of nitrobenzene (200 mL) and acetic acid (300 mL), and was heated up to 120 °C. Then, newly distilled pyrrole (2.7 mL, 40 mmol) was added dropwise. The solution was stirred at 120 °C for 1 h and then cooled down to room temperature. It can be observed that the solution gradually changed from a yellow clear solution to a purple-black solution during the reaction. The resulting purple-black precipitate was filtered and washed several times with CH_3_OH (100 mL × 3). The product was further recrystallized twice with CH_3_OH and CHCl_3_ to finally obtained TPP4Br (3.44g, yield: 34%). ^1^H nuclear magnetic resonance (NMR) (CDCl_3_, 500 MHz): δ (ppm) = −2.90 (s, 2H, NH), 7.9 (d, 8H, J = 8.3 Hz, aromatics), 8.02 (d, 8H, J = 8.3 Hz, aromatics), 8.77 (s, 8H, H-pyrrole). ^13^C NMR (CDCl_3_, 500 MHz): δ (ppm) = 140, 135, 130, 122, 118. matrix assisted laser desorption ionization-time of flight mass spectrometry (MALDI-TOF MS): m/z calculated for C_44_H_26_Br_4_N_4_, 929.89; found, 930.87. Ultraviolet*–*visible (UV*–*vis) (λ; nm; CH_2_Cl_2_ solution): 368, 418, 514, 551, 591 and 646.

#### 2.2.2. Synthesis of Platinum (II) 5,10,15,20-tetrakis(4-bromophenyl)-21,22-dihydroporphyrin (Pt-TPP4Br)

Under N_2_ atmosphere, PtCl_2_ (136 mg, 0.51 mmol) in PhCN (10 mL) was stirred at 130 °C for 12 h. The solvent was removed by vacuum distillation to obtain PtCl_2_(PhCN)_2_, and then sodium propionate (19.3 mg, 0.2 mmol) and chlorobenzene were added and refluxed for 2 h. After the reaction, the product was directly poured onto a silica gel column and eluted with CHCl_3_. The solvent was removed by distillation under reduced pressure to obtain a crude product. Then acetonitrile and 2 drops of concentrated HCl were added thereto, and the resulting suspension was stirred at 70 °C for 1 h, and then cooled to room temperature. After filtration, the residue was washed with a large amount of acetonitrile, and dried to give a brown-red product (denoted as Pt-TPP4Br, 31 mg, yield: 69%). ^1^H NMR (500 MHz, CDCl_3_): δ (ppm) = 8.77 (s, 8H, PorH_β_), 8.02 (d, J = 8.3 Hz, 8H, ArH), 7.9 (d, J = 8.3 Hz, 8H, ArH). ^13^C NMR (CDCl_3_, 500 MHz,): δ (ppm) = 140.8 (Cq), 139.9 (Cq), 135 (CH), 130.8 (CH), 130.1 (CH), 122.8 (Cq), 121.2 (Cq). MALDI-TOF MS: m/z calculated for C_44_H_24_Br_4_N_4_Pt, 1122.99; found, 1123.8. UV*–*vis (λ; nm; CH_2_Cl_2_ solution): 402, 509 and 538.

#### 2.2.3. Synthesis of Pt Porphyrin-based Conjugated Microporous Polymers (CMP-PtN4)

Refer to the literature and make appropriate adjustments. The solution of cod (0.127 mL, 1.04 mmol), [Ni(cod)_2_], (0.264 g, 0.96 mmol) and 2,2′-bipyridyl (0.15 g, 0.96 mmol) in anhydrous DMF (80 mL) was heated at 80 °C for 40 min. The previously obtained Pt-TPP4Br (225 mg, 0.2 mmol) was dissolved in anhydrous DMF (40 mL) and injected into the above purple solution. The mixture was stirred at 120 °C for 3 days under N_2_ atmosphere. The dark brown suspension was cooled to room temperature and 1 M HCl (400 mL) was injected and stirred for 0.5 h. After filtration, the residue was washed several times with CHCl_3_ (5 × 30 mL), THF (5×30 mL) and H_2_O (5 × 30 mL), and then dried under vacuum to obtain a brown powder (denoted as CMP-PtN4) in 85% yield. Fourier transform infrared (FTIR) (ν; cm^−1^): 1630, 1383, 1079 (υ_as_ (C-Br), weak), 1015 (δ_Pt–N_), 798, 709.

#### 2.2.4. Pyrolysis of CMP-PtN4

CMP-PtN4 (50 mg) was loaded into tube furnace, and subsequently heated to T °C for 2 h under N_2_ atmosphere under the heating rate of 5 °C min^−1^. After cooling to room temperature, the products were ground into fine powders (denoted as PC-PtN4-T, T = 600, 900).

### 2.3. Characterization

All NMR spectroscopy were measured on a Bruker 500 (500 MHz) spectrometer (Bruker, Karlsruh, Germany) for ^13^C and ^1^H. MALDI-TOF MS was carried out on an autoflex speed TOF/TOF instrument (Bruker, Karlsruhe, Germany). FTIR spectroscopy (Perkin Elmer, Boston, MA, USA) was performed on a Spectrum 100 instrument. UV*–*vis spectroscopy data were acquired on Lamda 950 instrument (Thermo Fisher, Waltham, MA, USA). X-ray diffraction (XRD) measurements were performed on a D8 Advance X-ray diffractometer (Bruker, Karlsruhe, Germany) with a scanning speed of 6° min^−1^ over the range 20–70° (2θ) with Cu Kα radiation (k = 1.54 Å) as the incident beam at a generator voltage of 40 kV and a generator current of 50 mA. Scanning electron microscopy (SEM, FEI, Portland, OR, USA) images were measured on a FEI Nova NanoSEM 450 instrument. Transmission electron microscopy (TEM, FEI, Portland, OR, USA) images were acquired using a FEI Tecnai G2 F20 S-TWIN instrument operated at 200 kV. Thermogravimetric analysis (TGA, TA, New Castle, DE, USA) measurements were performed on a Discovery TGA550 instrument. X-ray photoemission spectroscopy (XPS, Shimadzu, Kyoto, Japan) were performed on an AXIS UltraDLD instrument. Solid-state NMR spectra measurements were performed on an AVANCE NEO SK2-2-12a spectrometer operating (Bruker, Karlsruhe, Germany) at 600.13 MHz for ^13^C NMR.

### 2.4. Electrochemical Measurements

The electrochemical experiments for HER were carried out in a conventional three-electrode cell using a CH Instrument (model 660D) at room temperature. Ag/AgCl (3 M KCl) and carbon rod were used as reference and counter electrodes, respectively. The preparation of catalyst slurry is in accordance with the rule of mixing 1 mg catalyst with 100 μL of Nafion solution (0.05 wt. %) [27]. Catalysts ink were prepared by blending 5 mg catalyst (CMP-PtN4, PC-PtN4-600, PC-PtN4-900, Pt/C, PC-PtN4-600/SP) with 500 μL of Nafion solution (0.05 wt. %) and stirred for 24 h. Especially, PC-PtN4-600 was mixed with Super P (SP) to obtain catalyst inks (PC-PtN4-600/SP (m/m, 1/1, 1/4, 1/8)). For working electrodes, the glassy carbon electrode surface (0.2471 cm^2^) was dropped with 9 μL of catalyst ink. Then the electrode with catalysts were dried at room temperature before testing. The polarization curves were obtained in 0.5 M H_2_SO_4_ with a scan rate of 10 mV s^−1^ at room temperature, 1600 rpm. All potentials in this study were iR compensated and referred to a reversible hydrogen electrode (RHE) via calibration measurement in N_2_-saturated electrolyte. EIS measurements were made at an open circuit voltage from 100 kHz to 0.01 Hz, with an amplitude of 10 mV. The double-layer capacitance (C_dl_) of the catalyst was calculated by CV curve, which were measured in the range of 0.3–0.4 V vs SCE at a scan rate of 20–200 mV s^−1^. The CV as a function of scan rate was plotted by the capacitor currents ΔJ (J_anodic_ − J_cathodic_). The C_dl_ was one-half of the slope of the fitting line, which is linearly proportional to the electrochemical active surface area of the electrode.

## 3. Results and Discussion

**Figure 1:**
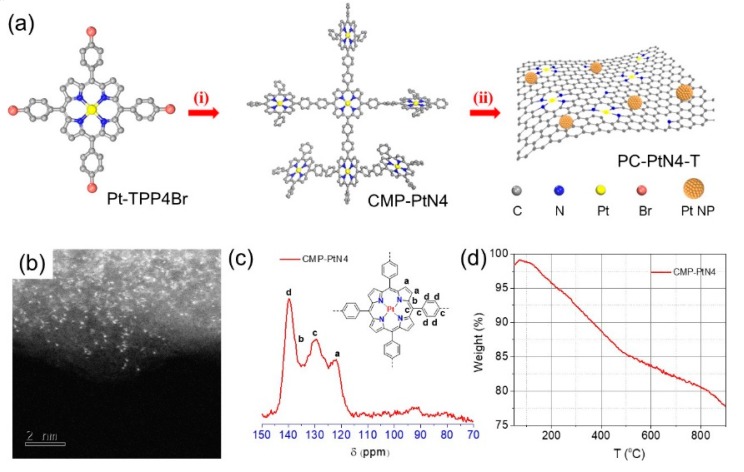
(**a**) Schematic representation of the synthesis route of PC-PtN4-T: (i) N_2_, Ni(cod)_2_, cod and 2,2′-bipyridyl, 80 °C, 3 days, (ii) N_2_, T °C (T = 600, 900), 2 h. HAADF-STEM image (**b**), ^13^C ssNMR spectrum (**c**), and TGA curve (**d**) of CMP-PtN4.

Figure 1a shows the synthesis of Pt single atoms and Pt ultra-fine NPs embedded porous carbons. At first, Pt porphyrin-based conjugated microporous polymer (denoted as CMP-PtN4) was synthesized by one-step Yamamoto polymerization of Pt(II) 5,10,15,20-tetrakis(4-bromophenyl)-21,22-dihydroporphyrin monomer (Pt-TPP4Br), which can be easily synthesized in large quantity [45]. The structure characterization Pt-TPP4Br was estimated by NMR spectroscopy, MALDI-TOF MS, UV-vis spectroscopy and FTIR spectroscopy (Scheme S1, Figures S1–S3). After pyrolysis of CMP-PtN4 at 600 or 900 °C for 2 h under inert atmosphere, Pt single atoms and Pt ultra-fine NPs embedded porous carbons (denoted as PC-PtN4-T, T = 600, 900) can be easily prepared. Such facile and efficient approach paves the way for the synthesis of metal atoms and NPs embedded hybrid materials.

The morphology, structure and thermostability of CMP-PtN4 were investigated by HAADF-STEM (Figure 1b), ^13^C solid-state NMR (ssNMR) spectroscopy (Figure 1c) and TGA (Figure 1d), respectively. Obviously, atomically dispersed Pt can be observed for the CMP-PtN4, implying the four N coordinated Pt in porphyrin structure was maintained after polymerization. For ^13^C ssNMR spectrum, the carbon signals centered at 122.2, 129.5, 137.3 and 139.8 ppm can be ascribed to the carbon atoms of porphyrin core and benzene linker, which is consistent with the reported metalloporphyrin-based porous polymers [46]. The FTIR peaks of CMP-PtN4 are similar to that of monomer, implying that the Pt(II) porphyrin structure remained after polymerization (Figure S4). The weight loss of CMP-PtN4 during the temperature rising can be attributed to the decomposition of porous polymers and rearrangement of the fragments [47]. Upon rising to 600 and 900 °C, 84% and 78% weight can be maintained, respectively, suggesting good thermostability and favorable carbon-precursor nature of CMP-PtN4.

**Figure 2:**
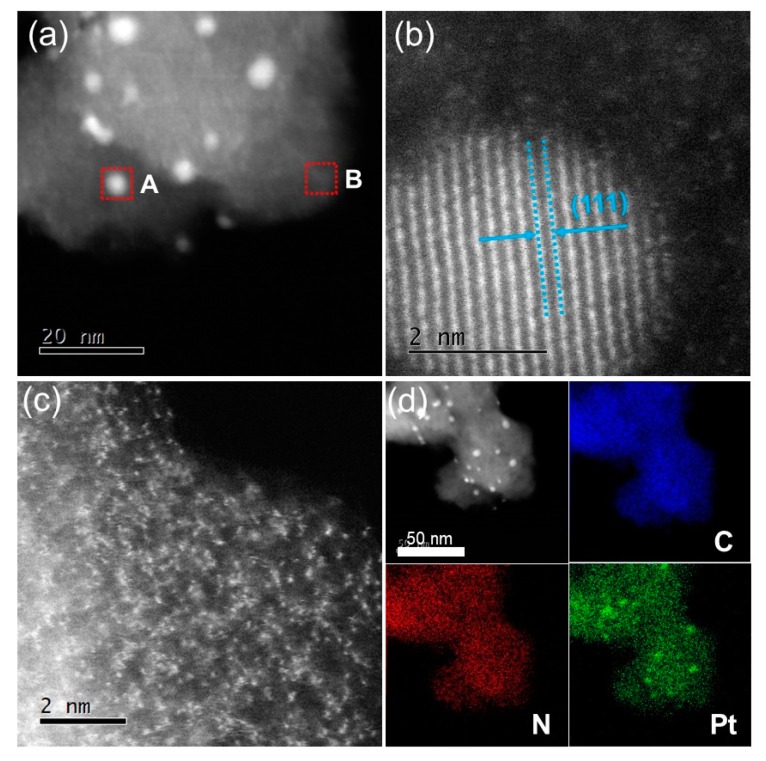
Morphology and elemental analysis of PC-PtN4-600. HAADF-STEM image (**a**) and high-resolution HAADF-STEM images acquired from the select areas A (**b**) and B (**c**) in (**a**). The HAADF-STEM image and corresponding elemental mapping of C, N, Pt (**d**).

The morphology and atomic-resolution structure of PC-PtN4-T are investigated by SEM, TEM, and HAADF-STEM. From the SEM images (Figure S5a–c), PC-PtN4-T are composed of irregular particles with submicron dimension, which inherited from CMP-PtN4 [48], but the carbon materials exhibit smoother surface [49]. From the TEM images (Figure S5d–f), numerous bright and dark areas can be observed for PC-PtN4-T and CMP-PtN4 [47], and the Pt NPs become bigger as the pyrolysis temperature rose from 600 to 900 °C. Taking the PC-PtN4-600 as an example, Pt NPs randomly disperse in the porous carbon with the size of several nanometers (Figure 2a). The high-resolution HAADF-STEM image of Pt NPs in PC-PtN4-600 demonstrates the crystalline nature of Pt NPs with typical (111) crystal plane of *fcc* Pt (Figure 2b) [50]. The high-resolution HAADF-STEM image of the areas without Pt NPs in PC-PtN4-600 show obvious atomically dispersed Pt (Figure 2c). Elemental mapping based on energy-dispersive X-ray analysis proves the uniform distribution of C and N in PC-PtN4-600 (Figure 2d). The homogenous dispersion of Pt in the areas without NPs demonstrates the existence of abundant atomically dispersed Pt in as-prepared porous carbon matrix. From the HAADF-STEM images of PC-PtN4-900 (Figure S6), Pt single atoms and Pt NPs embedded porous carbons can also be observed. The difference is that bigger Pt NPs can be found in PC-PtN4-900 than in PC-PtN4-600 because of the serious aggregation effect of Pt at high temperature.

**Figure 3:**
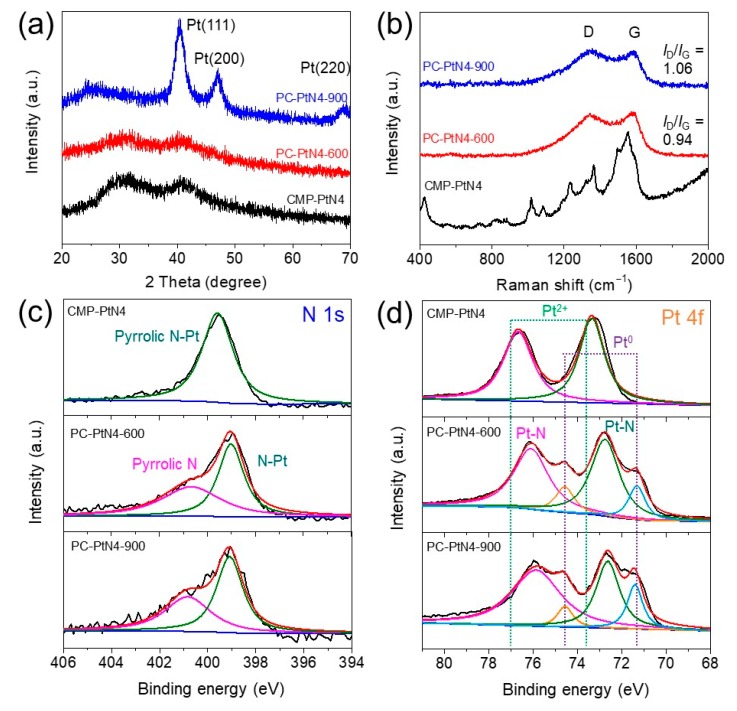
(**a**) X-ray diffraction (XRD) patterns, (**b**) Raman spectra, (**c**) N 1s X-ray photoemission spectroscopy (XPS) spectra and (**d**) Pt 4f XPS spectra of PC-PtN4-T.

The chemical structure and composition of PC-PtN4-T were further studied by XRD, Raman spectroscopy and XPS. XRD has been widely used to investigate the crystallinity of materials. As shown in Figure 3a, no obvious peaks corresponding to Pt crystal can be detected for PC-PtN4-600, suggesting the ultra-fine Pt nanoparticles in PC-PtN4-600 exceed the detection limit of XRD. For PC-PtN4-900, three peaks were observed to be located at 40.4°, 47.1° and 68.9°, which can be ascribed to the (111), (200), and (220) crystal planes of Pt NPs, respectively [51]. Such phenomenon implies the formation of large-sized Pt NPs in PC-PtN4-900, which is consistent with the morphology. Figure 3b shows the Raman spectra of as-prepared samples. The characteristic peaks of CMP-PtN4 located at the Raman shift of 426, 1018, 1084, 1236, 1363, 1497 and 1553 cm^−1^, are consistent with reported metalloporphyrin-based materials [52]. For PC-PtN4-T, two obvious broad peaks at 1347 and 1587 cm^−1^ can be observed, which are typical D-band and G-band of carbon materials. The *I*_D_/*I*_G_ value reflects the defect degree of the carbon materials. PC-PtN4-900 exhibits bigger *I*_D_/*I*_G_ value than that of PC-PtN4-600, implying more defect and worse conductivity of PC-PtN4-900 [53].

From the XPS survey spectrum of CMP-PtN4 (Figure S7a), only C, N, and Pt can be detected and no bromine signal was observed, suggesting the high polymerization degree of the porous polymers. The Pt content of CMP-PtN4 is estimated to be 18.77 wt. %. After pyrolysis, the Pt contents for PC-PtN4-600 and PC-PtN4-900 are about 18.70 wt. % and 18.94 wt. %, respectively (Figure S7b,c, Table S1), indicating low volatile feature of Pt under high temperature treatment. Figure 3c shows the N 1s XPS spectra of the samples. Only one N species can be found in CMP-PtN4, which is assigned to the internal pyrrolic N that connected to Pt. After pyrolysis, PC-PtN4-T exhibit two N species located at 400.7 and 399.0 eV, corresponding to pyrrolic N and the N associated with Pt (N-Pt), respectively, indicating partially decomposing of pyrrolic N-Pt bonds and N-doping into the carbon matrix [54,55]. The Pt 4f XPS spectra of the samples are shown in Figure 3d. The Pt in CMP-PtN4 displays typical oxidation state and the valence is close to +2, attributing to the Pt-N_4_ coordination environment originated from Pt porphyrin monomer. During the pyrolysis, part of Pt^2+^ atoms reduce to Pt^0^ and aggregate to form Pt NPs, while the rest of Pt^2+^ atoms reduce to lower oxidation valence because of the right shift of the binding energy peaks [40,51]. According to above results and HAADF-STEM images, as-prepared porous carbons are composed both of abundant N coordinated single Pt atoms and NPs.

**Figure 4:**
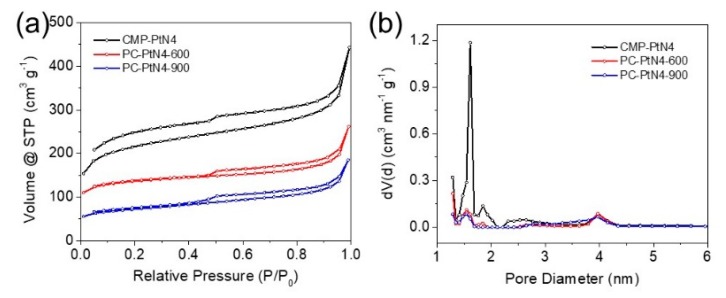
Nitrogen adsorption–desorption isotherms (**a**) and the corresponding pore size distributions (**b**) of CMP-PtN4, PC-PtN4-600 and PC-PtN4-900.

The porous nature of the samples is studied by nitrogen physisorption measurement. Figure 4a shows the nitrogen adsorption–desorption isotherms of as-prepared materials. Typical type IV curves with small H2 hysteresis loops for all the samples can be observed, implying the existence of cage-like mesoporous structure. The absorbed volume at low P/P_0_ is positively related the contents of micropores and specific surface area (SSA) of the porous materials [56]. Consistent with this, the Brunauer–Emmett–Teller (BET) SSAs of CMP-PtN4, PC-PtN4-600 and PC-PtN4-900 are calculated to be 681, 429, and 236 m^2^ g^−1^, respectively (Table S2). The collapse of micropores during the pyrolysis is responsible for the reduced SSAs of the PC-PtN4-T. The pore size distributions calculated by nonlocal density functional theory (NL-DFT) model are shown in Figure 4b. Both micropores (pore diameter less than 2 nm) and mesopores (pore diameter between 2 and 50 nm) can be observed and the average pore sizes for CMP-PtN4, PC-PtN4-600 and PC-PtN4-900 can be calculated to be 2.9, 3.9, and 3.5 nm, respectively. The nitrogen physisorption properties of the samples are summarized in Table S2.

**Figure 5:**
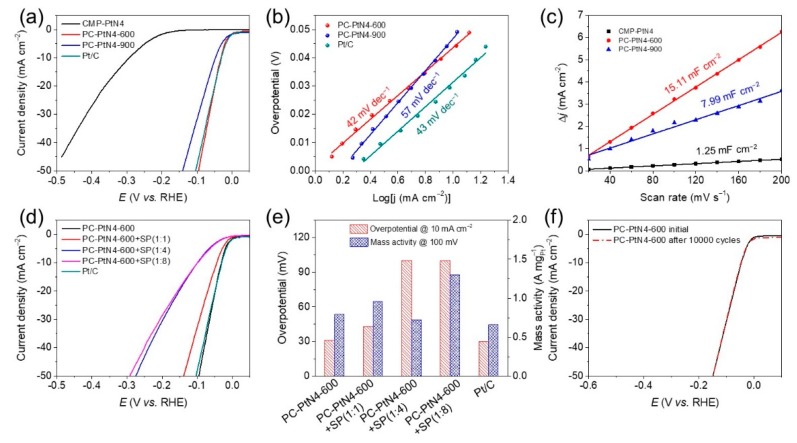
Electrocatalytic performance of the samples in 0.5 M H_2_SO_4_ under N_2_ at room temperature. (**a**) hydrogen evolution reaction (HER) polarization curves, (**b**) Tafel slope plots acquired from polarization curves, and (**c**) capacitive current at 0.35 V as the function of scan rate of CMP-PtN4, Pt/C and PC-PtN4-T. (**d**) HER polarization curves, (**e**) overpotential at 10 mA cm^−2^ and mass activity at 100 mV of PC-PtN4-600 and Super P carbon based composites. (**f**) Stability test of PC-PtN4-600. The polarization curves were recorded initially and after 10,000 potential cycles between −0.15 and + 0.15 V (vs. RHE) at 100 mV s^−1^.

The electrocatalytic performance of the PC-PtN4-T, together with CMP-PtN4 and commercial Pt/C were tested by using three-electrode setup with 0.5 M H_2_SO_4_ electrolyte under N_2_ atmosphere at room temperature. Figure 5a shows the HER polarization curves of the electrocatalysts. The PC-PtN4-T and Pt/C exhibit the lowest onset potential of 0 mV, while that of the CMP-PtN4 is 143 mV. The overpotentials of PC-PtN4-600 and Pt/C at 10 mA cm^−2^ are 31 and 30 mV, respectively, which are superior than that of PC-PtN4-900 (47 mV) and CMP-PtN4 (293 mV). PC-PtN4-600 exhibits comparable HER performance to Pt/C, it is attributed to the synergistic effect of the Pt atoms and ultra-fine NPs which lowers the kinetic energy barrier of the HER [20], as well as the favorable electronic conductivity and porosity. The reduced performance of PC-PtN4-900 is due to the aggregation of ultra-fine Pt NPs and thereby lower the Pt active sites, while CMP-PtN4 suffers from the poor electronic conductivity of the polymer backbone (Figure S8). In order to testify the catalytic activity derived from Pt species, Pt-free sample was prepared from the pyrolysis of porphyrin-based CMP (PC-N4-600), and then the HER activity was tested (Figure S9), suggesting the essential of Pt to achieve high performance of PC-PtN4-600. Tafel slope is an efficient way to investigate the intrinsic reaction kinetics of the catalysts for HER [57,58]. As shown in Figure 5b, PC-PtN4-600 displays a Tafel slope of 42 mV dec^−1^, which is very similar to that of Pt/C (43 mV dec^−1^), implying both of them share the same rate-determining step, where two hydrogen intermediates desorb and form H_2_ molecule (Tafel step). In contrast, PC-PtN4-900 shows a higher Tafel slope of 57 mV dec^−1^, indicating the slow reaction kinetic.

The double-layer capacitance (C_dl_) can be calculated from cyclic voltammetry (CV) measurement under different scan rates in specific voltage region, and further used to assess the effective surface active areas of the electrocatalysts [27]. Figure 5c shows the C_dl_ of as-prepared catalysts calculated from Figure S10. PC-PtN4-600 shows C_dl_ of 15.11 mF cm^−2^, which is 1.9 and 12.1 times higher than those of PC-PtN4-900 (7.99 mF cm^−2^) and CMP-PtN4 (1.25 mF cm^−2^), respectively. These results demonstrate higher effective surface active area of PC-PtN4-600 than those of PC-PtN4-900 and CMP-PtN4.

To further explore the catalytic ability of the catalyst, PC-PtN4-600 was mixed with SP in different ratios and then used as catalysts for HER performance evaluation (Figure 5d,e). The overpotentials at 10 mA cm^−2^ for the PC-PtN4-600 mixed with SP in 1/1, 1/4 and 1/8 are 45, 99 and 99 mV, respectively. The mass activity of PC-PtN4-600 and SP mixtures at 100 mV is up to 1.3 A mg_Pt_^−1^, which is twice of Pt/C (0.66 A mg_Pt_^−1^). Although the overpotential increased with the reduced content of PC-PtN4-600, but the mass activity of the catalyst was enhanced. The stability performance of PC-PtN4-600 was first studied by the accelerated degradation test (ADT) [57]. Typically, PC-PtN4-600 was sprayed on carbon paper and then the CV measurements was performed in N_2_-saturated 0.5 M H_2_SO_4_ electrolyte for 10,000 cycles between −0.15 to +0.15 V (vs. RHE). The polarization curves of the initial catalyst and the catalyst after 10,000 CV cycling are shown in Figure 5f. Obviously, PC-PtN4-600 shows excellent stability due to no obvious activity loss even after 10,000 CV cycles. Furthermore, another 20 h chronoamperometry measurement also demonstrated the robustness nature of PC-PtN4-600 under testing conditions (Figure S11).

**Figure 6:**
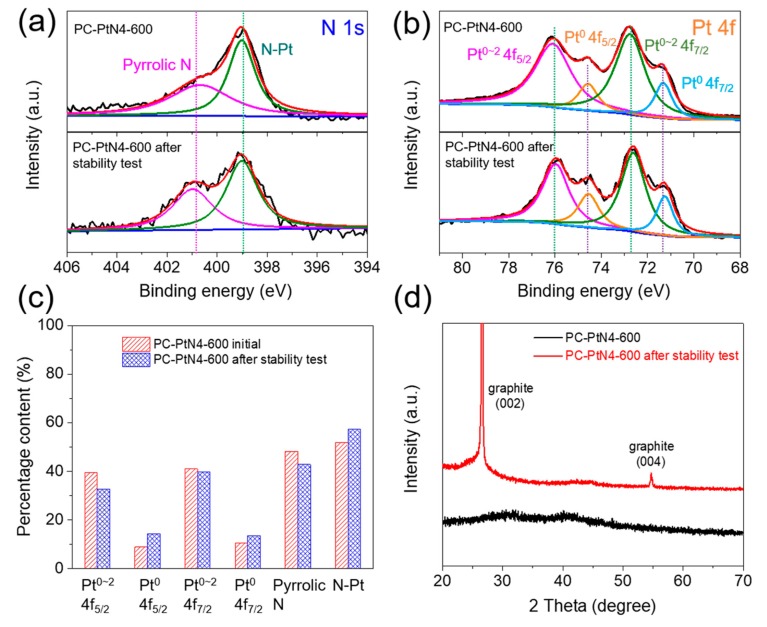
(**a**) N 1s XPS spectra, (**b**) Pt 4f XPS spectra and (**c**) Pt and N contents and (**d**) XRD patterns of PC-PtN4-600 before and after accelerated degradation test (ADT).

PC-PtN4-600-covered carbon paper electrode after ADT was recovered and studied by XPS (Figure 6a–c and Figure S12), XRD (Figure 6d) and HAADF-STEM (Figure S13). These measurements are sensitive to slight structure changes. By comparison with the characterization of initial PC-PtN4-600 before CV cycling, the N 1s and Pt 4f XPS spectra are shown in Figure 6a,b. Obviously, neglect binding energy change can be found for both the N and Pt fitted peaks, implying the type of N and Pt species is identical between initial and the cycled catalyst. Meanwhile, the contents of N and Pt species are calculated and summarized in Figure 6c. The similar values between the initial and cycled catalysts indicate the high stability of PC-PtN4-600. The XRD patterns (Figure 6d) and HAADF-STEM image (Figure S13) of the initial and cycled catalysts demonstrate the stability of the ultra-fine Pt NPs in PC-PtN4-600 which are not going to aggregate during the harsh electrochemical test. All in all, PC-PtN4-600 displays ultrahigh structure stability in acid electrolyte which ensures future practical application in water splitting.

## 4. Conclusions

In this work, an efficient HER electrocatalyst that constructed of atomically dispersed Pt and ultra-fine NPs embedded in N-doped porous carbon was prepared by pyrolysis of Pt porphyrin-based porous polymers at 600 °C. As-prepared PC-PtN4-600 exhibits dramatically enhanced activity with extremely low overpotentials of 31 mV at 10 mA cm^−2^. Moreover, its mass activity is up to 1.3 A mg_Pt_^−1^ at overpotential of 100 mV, which is two times larger than that of commercial Pt/C (0.66 A mg_Pt_^−1^). This favorable HER performance can be ascribed to the synergistic effect of the Pt atoms and ultra-fine NPs which lowers the kinetic energy barrier of the HER, as well as the enough electronic conductivity and abundant effective active sites. Such a new strategy of preparing N-doped porous carbons with both atomically dispersed metal active sites and corresponding metal NPs is a universal method, and can be expanded to other metals and provides diverse electrocatalysts for energy conversion beyond HER, such as oxygen reduction reaction, carbon dioxide reduction, etc.

## Supplementary Materials

The following are available online at https://www.mdpi.com/1996-1944/13/7/1513/s1, Scheme S1: Synthesis route to Pt-TPP4Br. (i) nitrobenzene and acetic acid; (ii) PtCl_2_, PhCN, Figure S1: ^1^H NMR spectra of TPP4Br (a) and Pt-TPP4Br (b), Figure S2: ^13^C NMR spectra of TPP4Br (a) and Pt-TPP4Br (b), Figure S3: UV-vis spectra (a) and FTIR spectra (b) of TPP4Br and Pt-TPP4Br, Figure S4: Comparison of the FTIR spectra of Pt-TPP4Br and CMP-PtN4, Figure S5: SEM images (a, b, c) and TEM images (d, e, f) for CMP-PtN4, PC-PtN4-600, and PC-PtN4-900, respectively, Figure S6: Morphology and elemental analysis of PC-PtN4-900. HAADF-STEM image (a). High-resolution HAADF-STEM images acquired from the select area A (b) and B (c) in (a). The HAADF-STEM image and corresponding elemental mapping of C, N, Pt (d), Figure S7: XPS survey (a, b, c) and C 1s XPS spectra (d, e, f) for CMP-PtN4, PC-PtN4-600, and PC-PtN4-900, respectively, Figure S8: Nyquist plots of CMP-PtN4, PC-PtN4-600, and PC-PtN4-900, Figure S9: HER polarization curves of PC-PtN4-600 compared with PC-N4-600, Figure S10: CV curves of CMP-PtN4 (a), PC-PtN4-600 (b), and PC-PtN4-900 (c) with different scan rates from 20 to 200 mV s^−1^ in the region of 0.3–0.4 V vs SCE, Figure S11. The time-dependent current density curve of PC-PtN4-600 was obtained at a constant overpotential of η = 31 mV for 20 h under acidic conditions (0.5 M H_2_SO_4_), Figure S12: XPS survey (a) and C 1s XPS spectrum (b) of PC-PtN4-600 after stability test, Figure S13: HAADF-STEM image of PC-PtN4-600 after ADT. Table S1: C, N and Pt content of the as-prepared samples calculated by XPS, Table S2: Textural parameters of the as-prepared samples based on nitrogen physisorption.
