# Supplementary material for: Platinum Atoms and Nanoparticles Embedded Porous Carbons for Hydrogen Evolution Reaction
Source: Materials (Basel). 2020 Mar 26;13(7):1513. doi: 10.3390/ma13071513 (PMC7177507; doi:10.3390/ma13071513)
Supplement: Supplementary file 1 [file materials-13-01513-s001.pdf]

materials

MDPI

Article

Platinum Atoms and Nanoparticles Embedded Porous Carbons for Hydrogen Evolution Reaction

Jialing Kang [1,2], Mengjia Wang [3], Chenbao Lu [2], Changchun Ke [4], Pan Liu [3], Jinhui Zhu [2,]* and Feng Qiu [1,]*and Xiaodong Zhuang [2,]*

1. School of Chemical and Environmental Engineering, Shanghai Institute of Technology, 201418 Shanghai, China; 176061210@mail.sit.edu.cn(J.K.)
2. The Meso-Entropy Matter Lab, State Key Laboratory of Metal Matrix Composites, Shanghai Key Laboratory of Electrical Insulation and Thermal Ageing, School of Chemistry and Chemical Engineering, Shanghai Jiao Tong University, 200240 Shanghai, castle@sjtu.edu.cn (C.L.)
3. School of Materials Science and Engineering, Shanghai Jiao Tong University, 200240 Shanghai, China; wmj10063@sjtu.edu.cn (M.W.); panliu@sjtu.edu.cn (P.L.)
4. School of Mechanical Engineering, Shanghai Jiao Tong University, 200240, Shanghai, China; kechangchun@sjtu.edu.cn (C.K.)

* Correspondence: zhujinhui1109@sjtu.edu.cn (J.Z.); fengqiu@sit.edu.cn (F.Q.); zhuang@sjtu.edu.cn (X.Z.)

Received: 20 February 2020; Accepted: 23 March 2020; Published: 8 April 2020

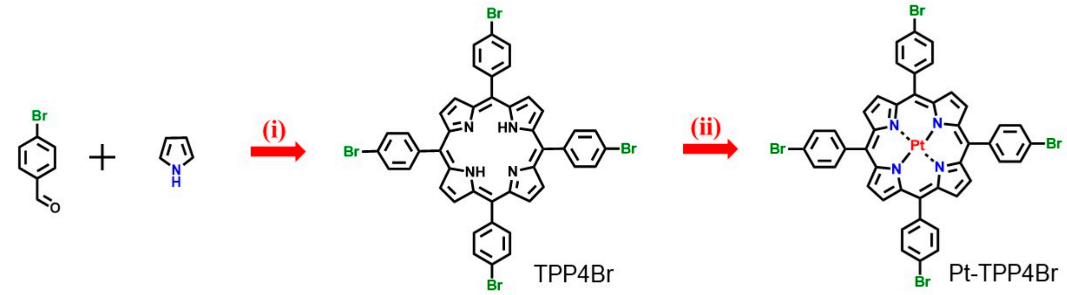

Scheme S1. Synthesis route to Pt-TPP4Br. (i) nitrobenzene and acetic acid; (ii) $PtCl_2$, PhCN.

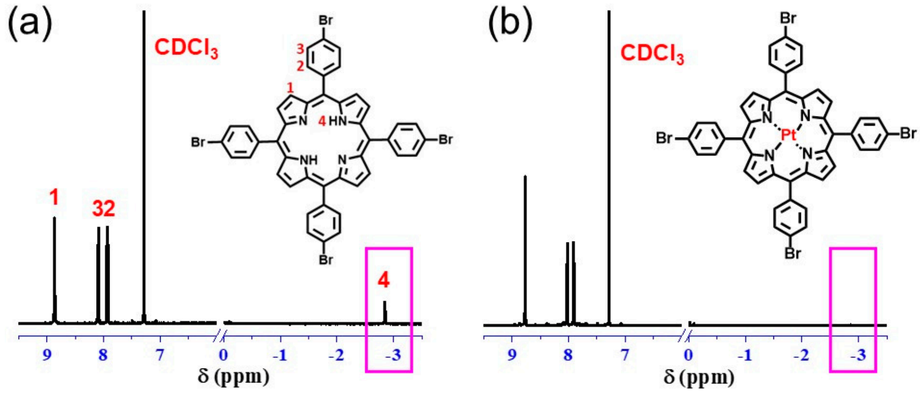

Figure S1. ^{1}H NMR spectra of TPP4Br (**a**) and Pt-TPP4Br (**b**).

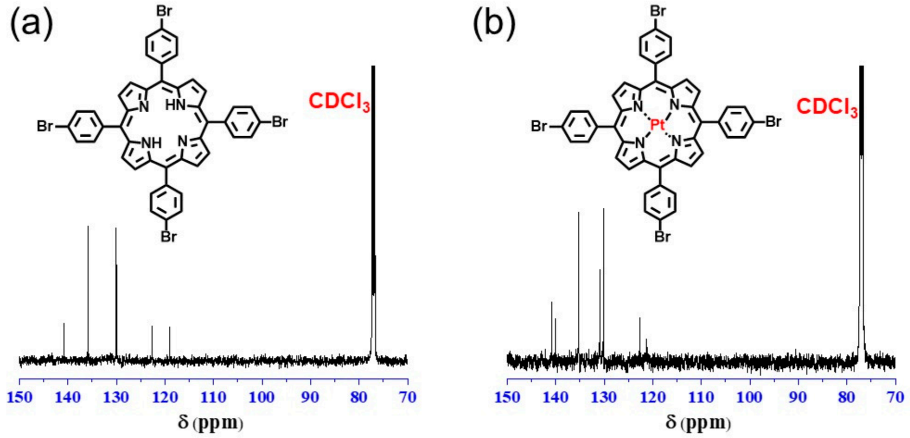

Figure S2. ^{13}C NMR spectra of TPP4Br (**a**) and Pt-TPP4Br (**b**).

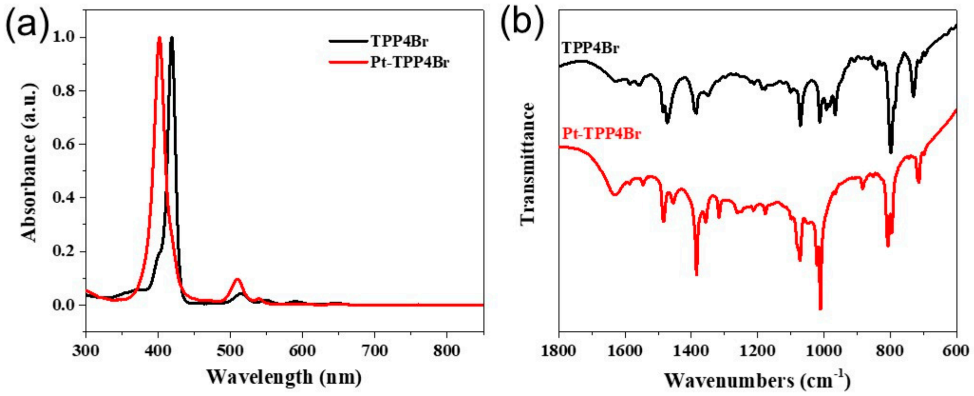

Figure S3. UV-vis spectra (**a**) and FTIR spectra (**b**) of TPP4Br and Pt-TPP4Br.

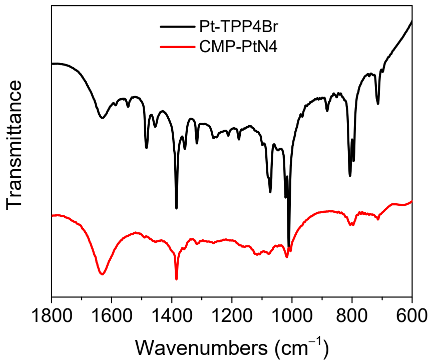

Figure S4. Comparison of the FTIR spectra of Pt-TPP4Br and CMP-PtN4.

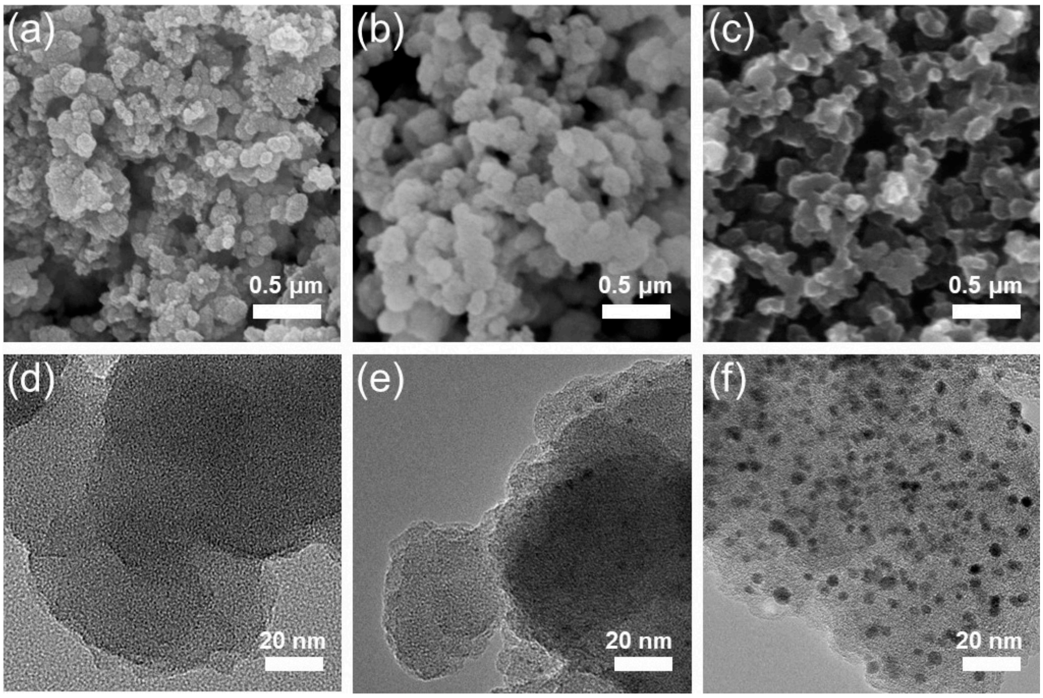

Figure S5. SEM images (**a**, **b**, **c**) and TEM images (**d**, **e**, **f**) for CMP-PtN4, PC-PtN4-600, and PC-PtN4-900, respectively.

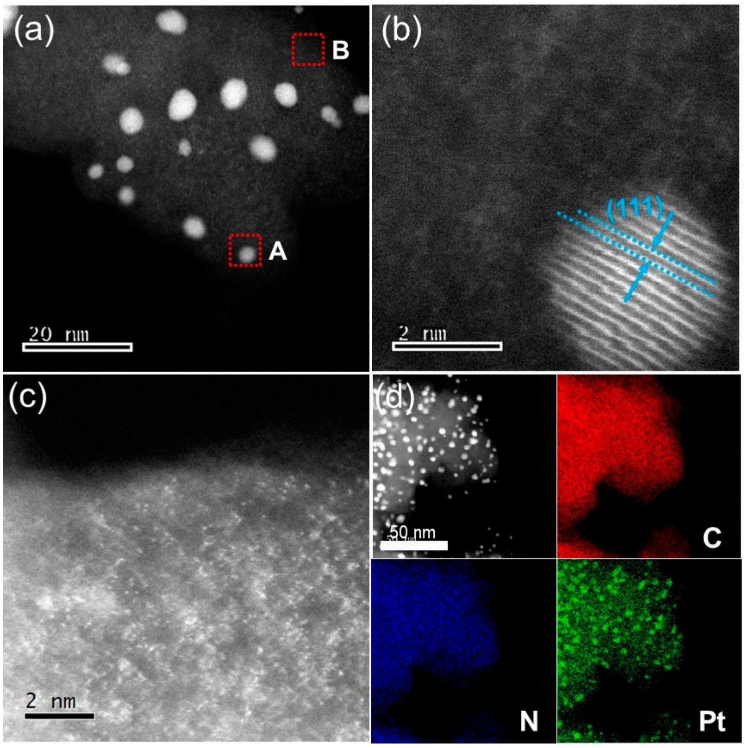

Figure S6. Morphology and elemental analysis of PC-PtN4-900. HAADF-STEM image (**a**). High-resolution HAADF-STEM images acquired from the select area A (**b**) and B (**c**) in (**a**). The HAADF-STEM image and corresponding elemental mapping of C, N, Pt (**d**).

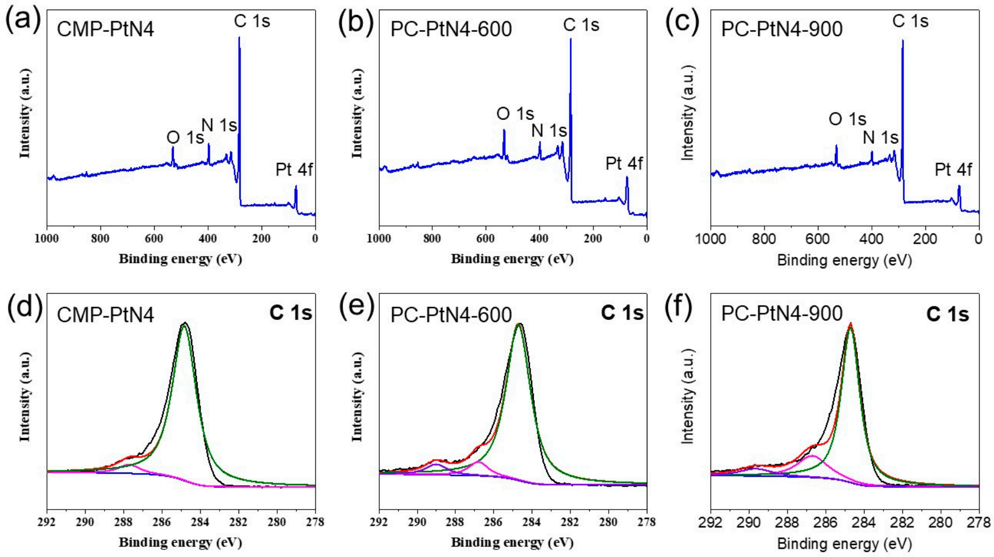

Figure S7. XPS survey (**a**, **b**, **c**) and C 1s XPS spectra (**d**, **e**, **f**) for CMP-PtN4, PC-PtN4-600, and PC-PtN4-900, respectively.

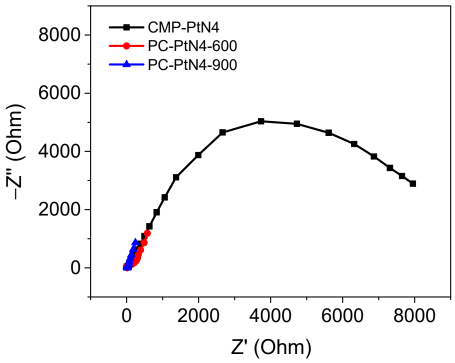

Figure S8. Nyquist plots of CMP-PtN4, PC-PtN4-600, and PC-PtN4-900.

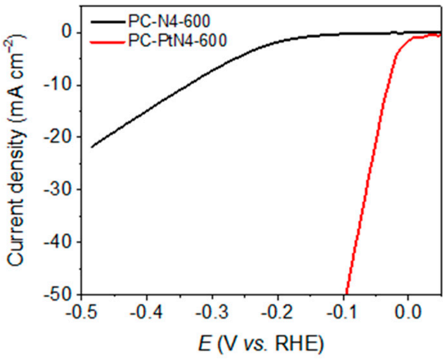

Figure S9. HER polarization curves of PC-PtN4-600 compared with PC-N4-600.

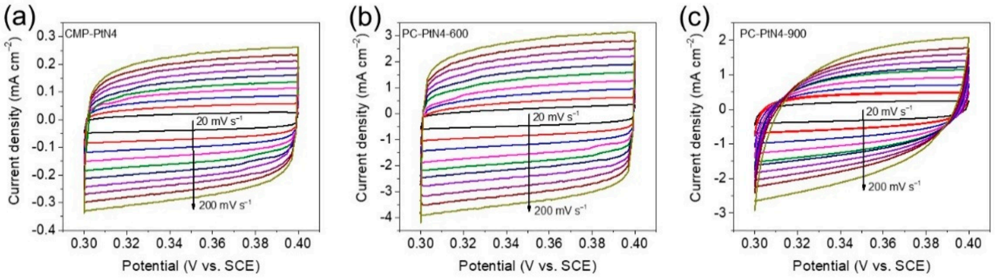

Figure S10. CV curves of CMP-PtN4 (**a**), PC-PtN4-600 (**b**), and PC-PtN4-900 (**c**) with different scan rates from 20 to 200 mV s^{-1} in the region of 0.3–0.4 V vs SCE.

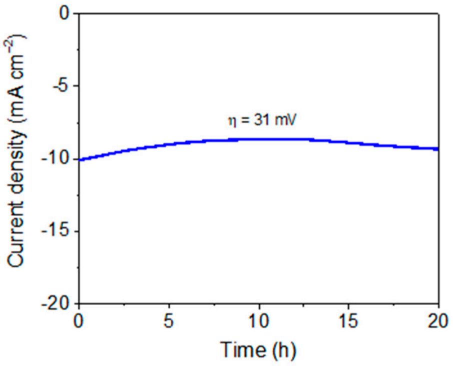

Figure S11. The time-dependent current density curve of PC-PtN4-600 was obtained at a constant overpotential of $\eta = 31$ mV for 20 h under acidic conditions (0.5 M H_2SO_4).

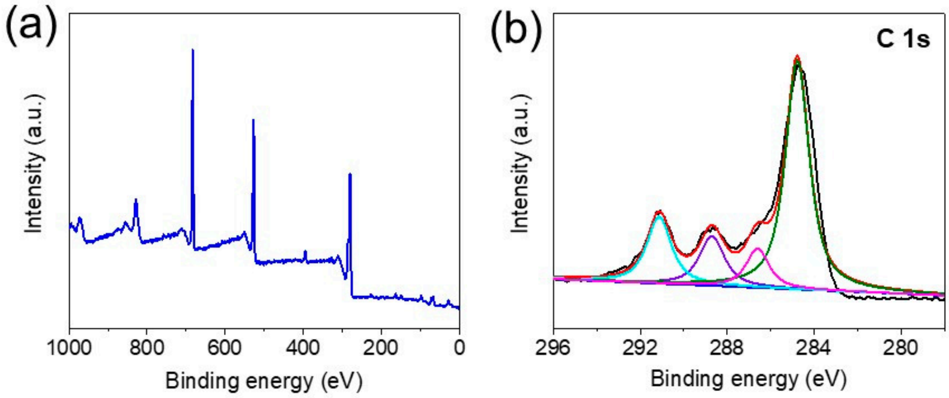

Figure S12. XPS survey (**a**) and C 1s XPS spectrum (**b**) of PC-PtN4-600 after stability test.

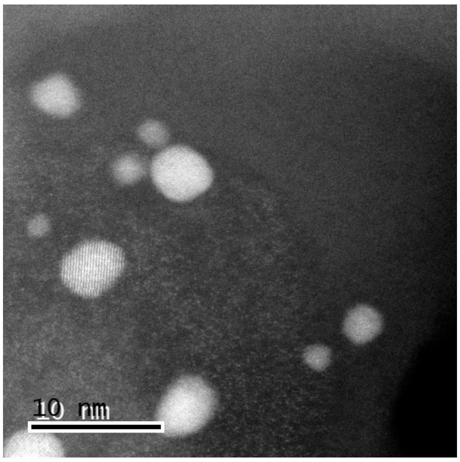

Figure S13. HAADF-STEM image of PC-PtN4-600 after ADT.

Table S1. C, N and Pt content of the as-prepared samples calculated by XPS.

Sample	C (wt.%)	N (wt.%)	Pt (wt.%)
CMP-PtN4	72.22	9.02	18.77
PC-PtN4-600	74.32	6.98	18.70
PC-PtN4-900	74.80	6.26	18.94

Table S2. Textural parameters of the as-prepared samples based on nitrogen physisorption.

Sample	S_{BET} (m^2 g^{-1})	S_{micro} (m^2 g^{-1})	V_{total} (cm^3 g^{-1})	V_{micro} (cm^3 g^{-1})	D (nm)
CMP-PtN4	681	531	0.50	0.29	2.9
PC-PtN4-600	429	371	0.24	0.19	3.9
PC-PtN4-900	236	152	0.22	0.08	3.5

© 2020 by the authors. Licensee MDPI, Basel, Switzerland. This article is an open access article distributed under the terms and conditions of the Creative Commons Attribution (CC BY) license (http://creativecommons.org/licenses/by/4.0/).
